# Impacts of Endogenous Factors and Ontogenetic Stages on the Metabolic Rate of the Mediterranean Spider Crab *Maja squinado* (Herbst, 1788)

**DOI:** 10.1093/iob/obaf027

**Published:** 2025-06-26

**Authors:** M-C Raffalli, J-J Filippi, J Bracconi, N Gattacceca, J-B Ronchi-Perfetti, A Crescioni, J-H Lignot, E D H Durieux

**Affiliations:** Université de Corse, Unité Mixte de Recherche CNRS 6134 SPE, Faubourg Saint-Antoine, 20250 Corte, France; Université de Montpellier, Unité Mixte de Recherche CNRS 9091 MARBEC, Pl. E. Bataillon, 34095 Montpellier, France; Université de Corse, Unité d'Appui et de Recherche CNRS 3514 STELLA MARE, Lido de la Marana, 20620 Biguglia, France; Université de Corse, Unité d'Appui et de Recherche CNRS 3514 STELLA MARE, Lido de la Marana, 20620 Biguglia, France; Université de Corse, Unité d'Appui et de Recherche CNRS 3514 STELLA MARE, Lido de la Marana, 20620 Biguglia, France; Université de Corse, Unité d'Appui et de Recherche CNRS 3514 STELLA MARE, Lido de la Marana, 20620 Biguglia, France; Université de Corse, Unité d'Appui et de Recherche CNRS 3514 STELLA MARE, Lido de la Marana, 20620 Biguglia, France; Université de Corse, Unité d'Appui et de Recherche CNRS 3514 STELLA MARE, Lido de la Marana, 20620 Biguglia, France; Université de Montpellier, Unité Mixte de Recherche CNRS 9091 MARBEC, Pl. E. Bataillon, 34095 Montpellier, France; Université de Corse, Unité Mixte de Recherche CNRS 6134 SPE, Faubourg Saint-Antoine, 20250 Corte, France; Université de Corse, Unité d'Appui et de Recherche CNRS 3514 STELLA MARE, Lido de la Marana, 20620 Biguglia, France

## Abstract

In the Mediterranean, populations of the spider crab *Maja squinado* are drastically declining. As a critical step toward restoration efforts, this study investigates ontogenetic metabolic changes from larvae to adults, accounting for size, molt stage, and sex. Routine metabolic rates were measured in reared larvae and juveniles, and wild-caught adults.

Zoea 1, the first planktonic stage, showed higher metabolic rates than zoea 2, likely due to a greater proportion of metabolically inactive tissue and differing energy sources (egg reserves vs. exogenous feeding). From megalopa to benthic juvenile stages, metabolic rates increased exponentially, probably reflecting increased organ complexity and activity. However, rates declined significantly from 7-month-old juveniles to adults, potentially due to reduced growth, longer intermolt periods, and behavioral adaptations. Among adults, males exhibited metabolic rates twice as high as females, likely linked to greater territorial and reproductive activity.

In 7-month-old juveniles at 14°C, mass-specific metabolic rate is inversely correlated with body size: individuals <20 g consumed oxygen at twice the mass-specific rate of those >80 g. The log_10_ of oxygen consumption positively correlated with log_10_ wet mass, with a “b” factor of 0.83. Molting also drastically influences metabolic activity, with lower rates observed in postmolt individuals than in individuals in premolt stages.

The successful rearing of *M. squinado* and the significant physiological insights gained into the different developmental stages enhance our understanding of the species' biological processes, and pave the way for further analyses before the implementation of restoration trials.

## Résumé (français)

En Méditerranée, les populations de l'araignée de mer *Maja squinado* sont en fort déclin. Dans le cadre des efforts de restauration, cette étude constitue une étape cruciale en explorant les variations métaboliques au cours du développement, des larves aux adultes, en tenant compte de la taille, du stade de mue et du sexe des individus. Les taux métaboliques de routine ont été mesurés chez des larves et juvéniles élevés en captivité, ainsi que chez des adultes capturés en milieu naturel.

Le stade Zoé 1, premier stade planctonique, présente des taux métaboliques plus élevés que le stade Zoé 2, probablement en raison d'une plus grande proportion de tissus métaboliquement inactifs et de sources d’énergie différentes (réserves vitellines vs. alimentation exogène). Du stade mégalope (planctonique) jusqu'aux stades juvéniles (benthiques), les taux métaboliques augmentent de façon exponentielle, reflétant vraisemblablement une complexification des organes et une activité accrue. En revanche, ces taux diminuent significativement entre les juvéniles de 7 mois et les adultes, ce qui pourrait s'expliquer par une réduction de la croissance, un allongement des périodes inter-mues et des adaptations comportementales.

Chez les adultes, les mâles présentent des taux métaboliques deux fois plus élevés que les femelles, probablement en lien avec une activité territoriale et reproductive plus intense.

Chez les juvéniles de 7 mois à 14°C, le taux métabolique est inversement corrélé à la taille corporelle : les individus de moins de 20 g consomment de l'oxygène par gramme de tissu frais à un taux deux fois supérieur à celui des individus de plus de 80 g. Le logarithme décimal de la consommation d'oxygène est positivement corrélé au logarithme de la masse fraîche, avec un facteur b de 0.83 (mâles et femelles confondus). La mue influence également fortement l'activité métabolique, avec des taux plus faibles observés chez les individus en post-mue que chez ceux en pré-mue.

Le succès de l’élevage de *M. squinado* et les enseignements physiologiques significatifs obtenus sur les différents stades de développement permettent une meilleure compréhension des processus biologiques de l'espèce et ouvrent la voie à de futures analyses avant la mise en œuvre d'essais de restauration.

## Introduction

Metabolism is the biological transformation of energy by organisms: resources are extracted from the environment and processed by individuals for various physiological functions such as survival, growth, and reproduction. Although these mechanisms vary greatly from one species to another, the underlying biochemical reactions are broadly similar across all organisms (Brown et al. 2004; Pettersen et al. 2016). This metabolic demand can play a fundamental role in the survival and behavior of organisms (Álvarez and Nicieza 2005; Biro and Stamps 2010; Larivée et al. 2010; Killen et al. 2011). The metabolic rate reflects the “pace of life” of organisms (Pettersen et al. 2016), and is thus the most commonly used indicator to describe the latter. For heterotrophic organisms, energy is obtained through the oxidation of carbon compounds; the metabolic rate of a given individual can be extrapolated, among other methods, from its respiration rate (Brown et al. 2004; Biro and Stamps 2010; Burton et al. 2011; Pettersen et al. 2016). This measurement is used in all research studies on metabolic rates, and is applicable to a wide range of organisms such as marine crustaceans (Cerezo Valverde et al. 2009), fish (Álvarez and Nicieza 2005; Killen et al. 2011; Chabot et al. 2016; Prinzing et al. 2023), insects (Schimpf et al. 2012), rodents (Konarzewski and Diamond 1995; Larivée et al. 2010), reptiles (Peterson et al. 1999), birds (Blackmer et al. 2005), and humans. For nontorpid animals, the standard metabolic rate (SMR) is the oxygen consumption rate of an organism that is postabsorptive or unfed, inactive, and free from anaerobic activity debt (Chabot et al. 2016). It represents the minimum amount of oxygen necessary for an organism to sustain its aerobic metabolic rate, and thus its vital functions. As the SMR is almost impossible to obtain without the use of pharmaceutical intervention to neutralize any voluntary movements, one approach is to measure the routine metabolic rate (RMR). The RMR includes a minor activity cost (e.g., very low amplitude movements by the animal to maintain its position in the metabolic chamber [MC]). Comparative interspecific studies of SMR or RMR have significant ecological importance but do not always identify the causal mechanisms behind the differences they observe. In contrast, intraspecific studies could be better suited to identifying the factors driving metabolic rate variability, notably through development (Burton et al. 2011; Konarzewski and Książek 2013).

Growth is often defined as the increase in size or cell number over time, with the key concept of positive energy balance (energy intake exceeding energy expenditure) (Evans 1998). The energy cost of growth can be defined by “the heat production which is due to biosynthesis” (Parry 1983). It can represent a significant portion of an organism's total metabolic rate: 30% in garter snakes, for example Konarzewski and Książek (2013) and between 17 and 29% of the metabolism of an “average” ectotherm population (Parry 1983). It is also well documented that the mass of individuals, whether or not they are growing, has a significant effect on their metabolic characteristics and could alone account for more than half of the interspecific variation in metabolic rate (Brown et al. 2004; Schimpf et al. 2012; Chabot et al. 2016; Prinzing et al. 2023). Kleiber's law theorized that the metabolic rate was proportional to body mass to the power of three-fourth (meaning that when the log mass increases by four times, the log metabolic rate increases by three times): this is the “b” factor. In reality, the many experimental studies carried out on hundreds of species ranging from plants to mammals have shown that this “b” factor varies inter- and intra-specifically, mostly from two-third to 1. This value has been found to be correlated with many aspects of species ecology, such as lifestyle and ecological niche (Da Silva et al. 2006; Killen et al. 2010; Glazier 2014; Glazier and Gjoni 2024). Metabolism also varies depending on sex, particularly in sexually mature adult individuals, and this variation can be observed through metabolic rate measurements (Blackmer et al. 2005; Cerezo Valverde et al. 2009). Finally, molting induces significant physiological changes in arthropods, potentially leading to considerable variability in energy balance between different molting stages (Penkoff and Thurberg 1982; Huang et al. 2015). Typically, crabs in intermolt stage C (the longest lasting stage) are actively moving and feeding while those in premolt stages (stages D0-D2) reduce their energy investments in activities other than the active preparation of their new internal cuticle (Lipcius and Herrnkind 1982; O'Halloran and O'dor 1988). Postmolt crabs (stages A and B) generally fill with water to enlarge their body size and harden their new cuticle.

**Fig. 1.**
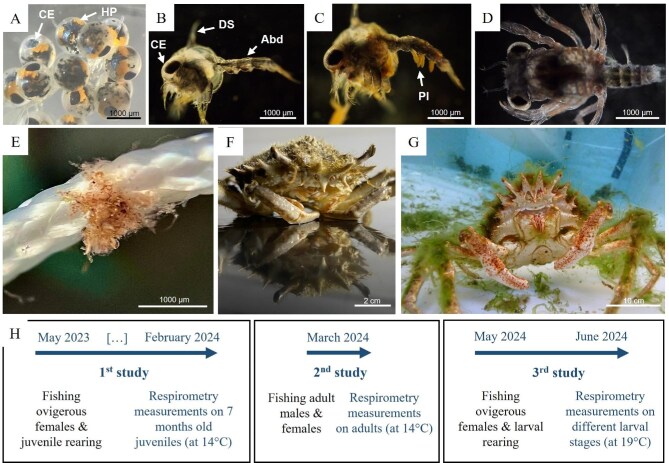
Pictures of different life stages of *Maja squinado*: (**A**) Eggs, (**B**) Zoea 1, (**C**) Zoea 2, (**D**) Megalopa, (**E**) C6 juvenile, (**F**) 7 months old juveniles, (**G**) Adult. (**H**) Timeline of capture for adult *Maja squinado* and of the three studies carried out between May 2023 and June 2024. CE: compound eye; HP: hepatopancreas; DS: dorsal spine; Abd: abdomen; Pl: pleopods.

The Mediterranean spider crab, *Maja squinado* (Herbst 1788), is a crustacean species within the Majidae family and has been genetically distinguished by Sotelo et al. (2008) from its Atlantic sister species, *Maja brachydactyla. Maja squinado* has not been classified by the International Union for Conservation of Nature (IUCN) despite the fact that it has either disappeared from its natural environment, as observed in the Balearic Islands (Durán et al. 2012), or, is in sharp decline. This decline could be partly explained by the species' low larval connectivity, particularly in the Balearic Islands, due to its short pelagic larval duration (Barrier et al. 2025). In Corsica, the Catch Per Unit Effort for *M. squinado* decreased fivefold between 2011 and 2020 in areas where this species was once abundant (Marengo et al. 2023). As an economically and ecologically important species throughout the Mediterranean Sea (United Nations Environment Program 2019), *Maja squinado* is an excellent candidate for ecological restoration (Durán et al. 2012; Guerao and Rotllant 2010). Repopulation trials have already been carried out in the Balearic Islands, namely at the Puerto Cristo marine reserve and Cabrera national park (JACUMAR 2009). Wild gravid females survive well in captivity and the larval phase (from egg hatching to the first juvenile stage C1) is rapid, lasting on average 17 days at 19.5°C (Durán et al. 2012). This larval phase is divided into three stages: zoea 1, zoea 2, and megalopa (Fig. 1). After reaching the postmetamorphic C1 stage, juveniles become benthic individuals and reach sexual maturity (i.e., become adults) after around 2 years. Despite the high potential of this species for aquaculture and restoration programs, there are, to our knowledge, no studies on its ecophysiology to date. Yet it is essential to understand the biological mechanisms of an organism (such as stress response mechanisms, growth cycles, reproductive cycles…) if we hope to better anticipate its interactions with its environment, and implement its ecological restoration. This study therefore aims to better describe the impact of intrinsic factors such as molt cycle stage, size, molt, and sex on the metabolic rate of *M. squinado*.

This study aims to unravel the driving factors for the variations in the metabolic rate of *M. squinado* throughout ontogenetic stages, along with key morpho-anatomical traits: size/mass changes, sex, and molting stage. It focuses on the metabolic rate of *M. squinado* individuals from the larval stages (zoea 1, 2, and megalopa) through juveniles (C1 and 7-month-old individuals) to fully mature adults.

## Material and methods

### Animal collection and rearing conditions

#### Adults

All the adult *Maja squinado* individuals (males and females) used for rearing and experimental purposes were captured in the northwestern Mediterranean Sea, along the Saint-Florent coast (Corsica, France). Individuals were caught by a local fisherman using a trammel net, at a depth of 30 m on a sandy-rocky seabed. To avoid thermal shock and stress, individuals were placed in a cooled icebox and transported directly to the STELLA MARE marine research center, arriving less than 3 h after coming ashore. All individuals were checked for anatomical integrity and no mortality was recorded. They were then transferred to individual square tanks (200 L) connected to a recirculated aquaculture system (RAS) under natural photoperiod. Water temperature was maintained constant: 19.15 ± 0.18°C for the ovigerous females (caught in May 2023 and 2024, Fig. 1) and 14 ± 0.22°C for nonreproducing adults used for respirometry analyses (caught in March 2024, Fig. 1). Water salinity was maintained at 37.5 g.kg^−1^ and water pH at 8 ± 0.09. Each day, individuals were fed *ad libitum* with a mixed diet of thawed squid, mussels, fish, and shrimp.

#### Larval rearing and juvenile maintenance

**Fig. 2.**
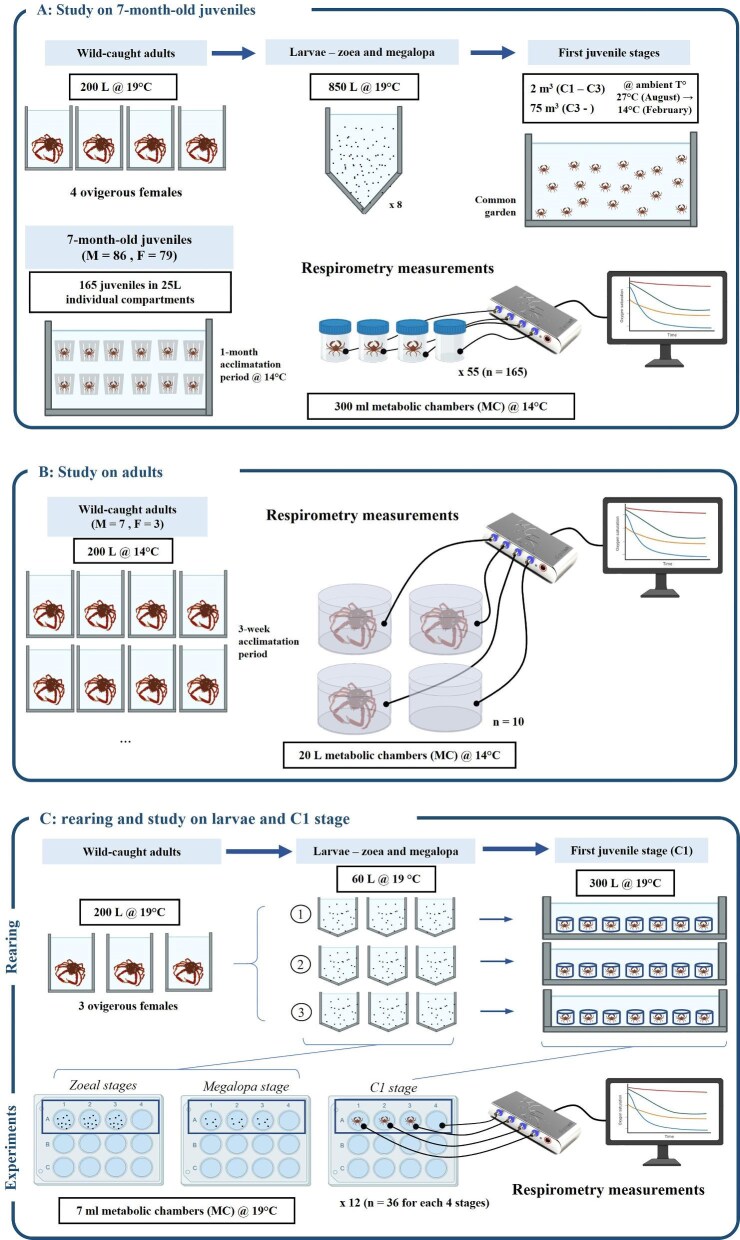
Study design for the 3 different measurements carried out between May 2023 and June 2024 on different developmental stages of *Maja squinado*: (A) 7-month-old juveniles; (B) adults; (C) larval stages and first juvenile stage (C1).

Newly hatched larvae that appeared active were distributed equally among the tanks but were placed in tanks containing different volumes of water: 850 L for larvae that will be reared to monitor oxygen consumption in juveniles (Fig. 2A), and 60 L for those destined for larval respirometry (Fig. 2C). Rearing conditions were identical for both batches. The initial rearing density was set at 70 larvae per liter of seawater. The temperature for all larval tanks was set at 19.15 ± 0.55°C, and water was sourced from another RAS system with similar characteristics. Salinity was maintained at 37.5 g.kg^−1^ and water pH at 8.02 ± 0.10. Larvae were fed with freshly hatched Artemia nauplii (*Artemia salina*) during the zoea stages then with metanauplii during the megalopa stage (INVE aquaculture, Dendermonde, Belgium), at a density of 4.3 *Artemia* per mL. On reaching the C1 stage (i.e., 18 days after hatching on average), the majority of these individuals were placed in a 2000 L outside tank, and the remainder were placed in individual compartments in the hatchery to avoid cannibalism, until the oxygen measurements had been carried out (Fig. 2C). They were fed daily *ad libitum* with HUFA SuperShrimp frozen enriched adult *A. salina* (Ocean Nutrition, Dartmouth, Canada). For C3 individuals and subsequent developing juveniles, individuals were placed in a 75 m^3^ outdoor raceway tank with continuous partial water replenishment (20% per hour of natural, filtered and disinfected seawater). They were fed every 2 days with a mixture of frozen mussels, prawns and fish. For individuals in outdoor raceways, the water temperature was the same as in the natural environment, averaging 27°C in August, then gradually declining to an average of 14°C in February.

### Study design

For the analysis on 7-month-old juveniles (first study 1, Fig. 1), four ovigerous females (mean weight: 1.464 ± 0.236 kg) were caught in May 2023. The eggs of these females hatched in captivity and ongrowing was carried out on larvae and juveniles. One hundred and sixty-five of these juveniles, weighing from 2.5 to 115 g (Table 1), were randomly collected in January 2024 and placed in individual 25 L oyster baskets (SEAPA, Nantes, France) in order to monitor mortality, growth and molting. In order to enable the individuals to acclimatize to their new environment, a 1-month acclimation period was implemented before starting the oxygen consumption measurements in February 2024. The measurements lasted 3 weeks. The water temperature was 14.5 ± 0.6°C (Fig. 2A). The animals were fasted for 3 days before measurement.

**Table 1.** Volume and number of individuals per MC, number of measures, body weight per individual, and sex ratio for all three studies on the spider crab *Maja squinado*

Study	S1	S2	S3			
Stage of individuals	7-month-old juveniles	Adults	Zoea 1	Zoea 2	Megalopa	C1
Volume of MC (mL)	300	13.000	7	7	7	7
Individuals per MC	1	1	10	10	5	1
Number of replicates	165	10	36	36	36	36
Body weight (mean ± SD) (g)	42.6 ± 24.7	1573 ± 195.8	0.65 × 10^−3^ ± 0.07^−3^	0.88 × 10^−3^ ± 0.09^−3^	1.5 × 10^−3^ ± 0.16^−3^	5.2 × 10^−3^ ± 1.06^−3^
Sex ratio (M/F)	86/79	7/3	––	––	––	––
CL (mean ± SD) (cm)	6.25 ± 1.47	––	––	––	––	––
Number of postmolting individuals	32	––	––	––	––	––

The analysis on adults (second study, Fig. 1) was conducted in March 2024 using adults placed in 200 L individual tanks. Seven males and three females (mean weight: 1.573 ± 0.196 kg) were used (Table 1). After a 3-week acclimation period, their oxygen consumption levels were measured over a 2-day period at a water temperature of 14 ± 0.4°C (Fig. 2B). The animals were fasted for 3 days before measurement.

For the analysis on larvae and the first juvenile stage (third study, Fig. 1), three ovigerous females (mean weight: 1.252 ± 0.358 kg) were caught in May 2024 and kept in captivity in 200 L separate tanks in order to collect newly hatched larvae (zoea 1). These larvae were placed in nine larval tanks (three tanks for the larvae of each female) in order to continue the rearing through their subsequent developmental stages (zoea 2, megalopa, and postmetamorphic C1 juveniles). For each of these stages in the larvae of each female, four independent measurements on different larvae were considered, with each measurement corresponding to three individual samples and one blank sample (four MC per measurement: MC 1 = larvae from tank 1, MC 2 = larvae from tank 2, MC 3 = larvae from tank 3 and MC 4 = blank). For one female and one stage, there are therefore four replicates per tank and 12 total replicates (three tanks with four replicates). Since three ovigerous females were used, this corresponds to 36 replicates per stage in total (Table 1). The water temperature was 19.1 ± 0.5°C (Fig. 2C). As the larvae and first juvenile stages were fed every day for rearing purposes, the measurements were taken just before feeding, that is, 24 h after the previous feeding.

### Indirect flow-through respirometry

Individual oxygen consumption rates were evaluated for the three studies at different developmental stages, namely larval stages to C1 juveniles, 7-month-old juveniles and wild-caught adults, using a FireSting O_2_ respirometer (Pyro Science, Aachen, Germany). This optical oxymeter was connected to a computer with the Pyro Oxygen Logger software on one side, and to four optical oxygen sensors (optodes) on the other, each of which was connected to the inner side of a MC. Throughout the analysis, the MCs were kept in an opaque tank and opaque lid to keep the animals in the dark. Oxygen concentration values, expressed as micromoles per liter, were directly sent to the software for data collection every second. Oxygen saturation was monitored for 1 h. For each batch of measurements, one of the four MCs contained water from the tank but no animals, in order to subtract the oxygen consumption by other potential organisms such as bacteria.

The volume capacity for the MCs must be selected according to the size of the animal analyzed. Volume capacity was thus 7 mL for larvae and C1 crabs (third analysis, Fig. 2C), 300 mL for 7-month-old juveniles (first analysis, Fig. 2A) and 20 L for adults (second analysis, Fig. 2B). The size of the MCs allowed only slight movement by individuals to ensure that the measured RMR was as close as possible to the SMR. The number of individuals in a MC was also dependent on the developmental stage: 10 individuals for zoea 1 and 2, 5 for megalopa (Fig. 2C), and 1 for juveniles and adults (Fig. 2A and B). After each measurement, biomass weight in each MC was measured using a VWR LA 614i weighing scale for larvae and juveniles (VWR, West Chester, USA) and an OHAUS ranger 7000 weighing scale for adults (Ohaus, Nänikon, Switzerland). For individuals from first and second studies (juveniles and adults), the length of the carapace (CL), that is, the distance between the rostral margin and the posterior margin of the carapace, was measured using a caliper, and individual sex was determined. For individuals from the first study (juveniles), the presence of a whole fresh molt in the individual basket was also recorded: these individuals are necessarily in the postmolt stage (A-B molting stages), the others being most likely in intermolt (molting stage C, lasting several weeks) but some may also be in premolt (molting stage D0–D2, lasting a few days). All these data are summarized in Table 1. The final oxygen consumption was calculated as follows:


\begin{eqnarray*}
{O}_2\, \textit{consumption} &=& \frac{{\left( {{{\left[ {{O}_{2}} \right]}}_{\textit{FINAL}} - {{\left[ {{O}_{2}} \right]}}_{\textit{INITIAL}}} \right) \times {\mathrm{\ }}V{\mathrm{\ }}}}{{\mathrm{W}}}\\ &&\quad -\, \textit{Blank}{\mathrm{\ }}\textit{consumption}
\end{eqnarray*}

**Fig. 3.**
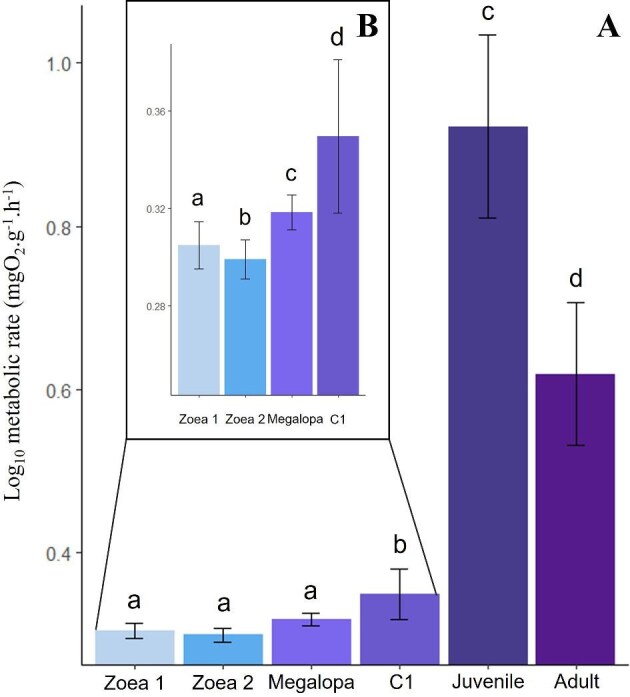
(**A**) Bar plots of the Log_10_ metabolic rate (mgO_2_.g^−1^.h^−1^) of the Mediterranean spider crab *Maja squinado*, according to developmental stage (zoea 1 and 2, megalopa, C1, juvenile and adult). (**B**) Insert detailing the data obtained on zoea 1 and 2, megalopa, and C1 stages. Mean ± standard deviation.

- V is the volume of the MC (in L),
- W is the weight of fresh material (in g),
- [O_2_] is the oxygen concentration in the MC (in mgO_2_.L^−1^),

We chose to present the values on Log_10_ scale in the graphs of Fig. 3, in order to minimize the visual gap between the larval/C1 stages and the juvenile/adult stages. The raw differences were otherwise too large, making the figure significantly less readable. However, in the written section of the results section, we retained the raw values to highlight the magnitude of these differences and to report the actual data.

### Statistical analysis

RStudio 2024.04.2 Build 764 software was used for all statistical analyses. Statistical comparisons between sexes (males and females) and molting stages (postmolting stage and pre- or inter-molting stages) were performed using independent Student *t*-tests, after checking the normality of the data using a Shapiro-Wilk test and the homogeneity of the variances using a Barlett test. Linear regressions (GLMM model) were used to assess the effect of individual body weight on oxygen consumption. To assess the oxygen consumption of individuals according to their life stage, one-way ANOVAs followed by post-hoc Tukey tests were carried out for all developmental stages (from zoea 1 to adults), then the same tests were repeated for the larval and first juvenile (C1) stages only. For larval and C1 stages, we first determined the impact of the maternal effect: we performed an ANOVA comparing the oxygen consumption of the 12 replicates of each female. The results showed *P*-values greater than 0.9. We concluded that no maternal effect was present and therefore carried out the following analyses without taking this factor into account.

## Results

All the results (statistical tests performed, *P*-values, degrees of freedom, F-factor, and adjusted R-squared) are detailed in Table 2.

**Table 2.** Statistical tests performed, *P*-values, degrees of freedom, F or t values, and adjusted R-squared, for each analysis

Groups tested	Test used	*P*-value(s)	D of freedom	F/t values	Adjusted R^2^
All stages (larvae to adults)	1 factor ANOVA	*P*-value <2.10^−16^	5	F:262.2	––
		Larvae/C1–juveniles: *P*-values <10^−7^			
	Tukey test	Larvae/C1–adults: *P*-values <0.01			
		Adults—juveniles: *P*-value <10^−7^			
Early stages (larvae-C1)	1 factor ANOVA	*P*-value = 5.7 × 10^−15^	3	F:31.97	––
	Tukey test	Zoea 1–zoea 2: 0.0381			
		Zoea 2–megalopa: 0.0057			
		C1–others: *P*-values <10^−7^			
Male/female (adults)	Independent student *t*-test	*P*-value = 0.0342	7.70	t:−2.56	––
Male/female (7-month-old juveniles)	Independent student *t*-test	*P*-value = 0.6014	149	t:−0.52	––
Postmolting stage/others (7-month-old juveniles)	Independent student *t*-test	*P*-value <10^−5^	133	t:4.66	––
Log_10_ dissolved O2 consumption/Log_10_ Body weight (7-month-old juveniles)	Linear regressions (GLMM model)	Males : *y* = 0.7699*x*–0.7419	77	F:465.6	0.8562
		*P*-value <2.2.10^−16^			
		Females: *y* = 0.8899*x*–0.9395	70	F:333.1	0.8239
		*P*-value <2.2.10^−16^			
Metabolic rate/body weight (7-month-old juveniles)	Linear regressions (GLMM model)	Males: *y* = −0.0007*x* + 0.1158	77	F:59.2	0.427
		*P*-value = 3.94.10^−11^			
		Females: *y* = −0.0004*x* + 0.0986	70	F:16.43	0.1785
		*P*-value = 0.0001			
Body weight/cephalothorax length (7-month-old juveniles)	Linear correlation	Pearson coefficient *r* = 0.95	157	––	––
		*P*-value <2.2.10^−16^			

### Oxygen consumption for different life stages (from zoea 1 to adult)

Dissolved oxygen consumption increased significantly across all ontogenetic stages (*P*-value <2e−16), with values increasing from 0.0005 ± 0.0001 to 0.0015 ± 0.0009 mgO_2_.g^−1^.h^−1^ between the first larval stage and the first juvenile stages (Fig. 3A). However, the increase was not continuous for the different larval stages (Fig. 3B), as the zoea 2 stage showed a significant slight drop (−14%) in metabolic rate compared with the zoea 1 stage (*P*-value = 0.0381). This was followed by a continuous significant increase for the megalopa and C1 stages (+58% and +110% respectively, *P*-values <10^−7^). The increase in metabolic rate was exponential in 7-month-old juveniles (*P*-value <10^−7^), rising from 0.0015 ± 0.0009 (C1 stage) to 0.0831 ± 0.0246 mgO_2_.g^−1^.h^−1^, a 55-fold increase (Fig. 3A). For adults, although oxygen consumption is much higher than the larval and C1 stages (*P*-values <0.01), it fell sharply compared with the levels in 7-month-old juveniles (loss of 69%) (*P*-value <10^−7^).

### Impact of biological factors on juvenile and adult O_2_ consumption rate

**Fig. 4.**
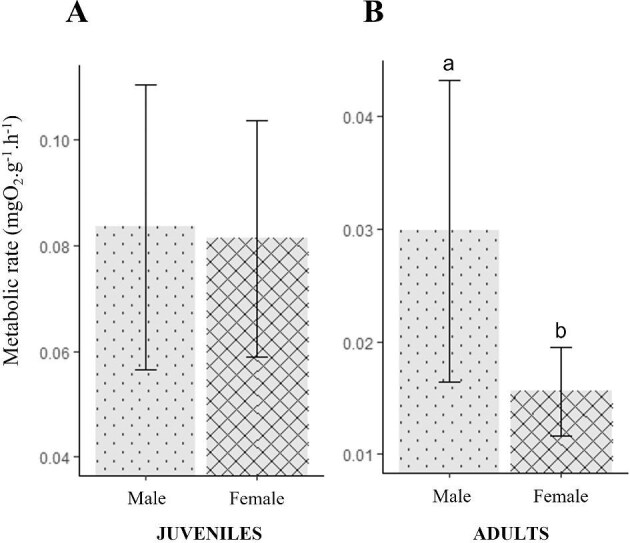
Bar plots of the metabolic rate (mgO_2_.g^−1^.h^−1^) of Mediterranean spider crab *Maja squinado* juveniles (**A**) and adults (**B**), in relation to their sex. Mean ± standard deviation.

In juvenile *M. squinado*, the sex of the individuals had no significant influence on the metabolic rate (Fig. 4A) (*P*-value = 0.6014): mean values are 0.0834 ± 0.0270 and 0.0813 ± 0.0223 mgO_2_.g^−1^.h^−1^ for males and females, respectively. However, O_2_ consumption per gram of fresh matter in adults was 50% lower in females than in males (*P*-value = 0.0342): mean values are 0.0298 ± 0.0134 and 0.0155 ± 0.0039 mgO_2_.g^−1^.h^−1^ for males and females, respectively (Fig. 4B).

**Fig. 5.**
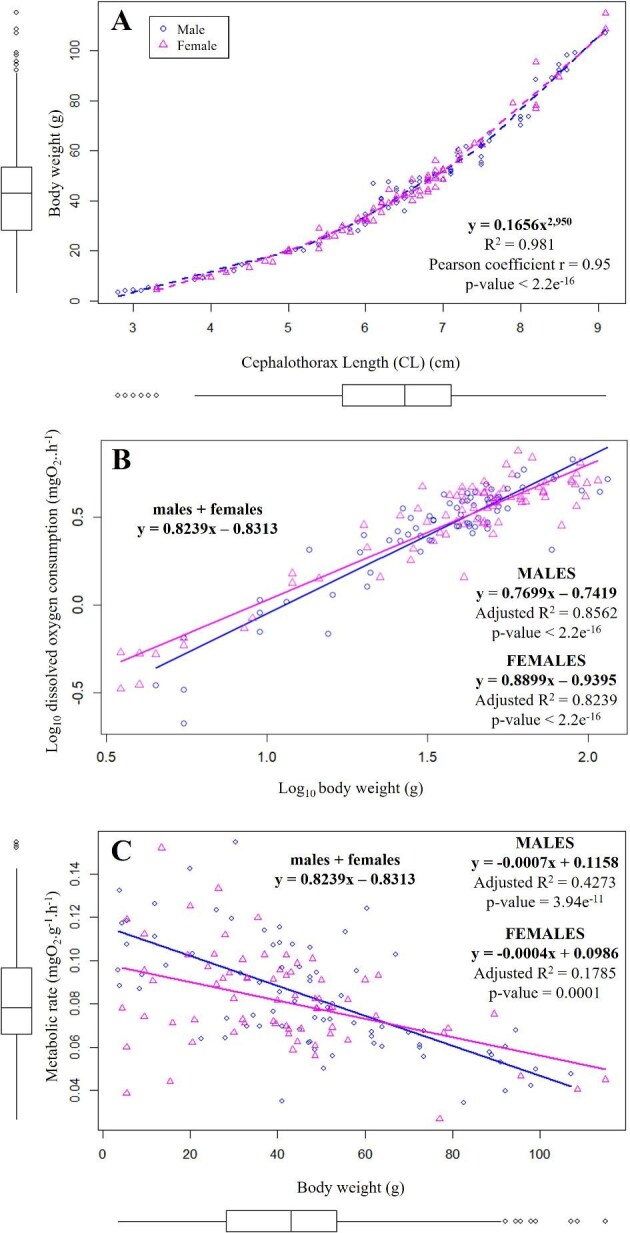
(**A**) Scatter plot of body weight (g) of Mediterranean spider crab *Maja squinado* juveniles, in relation to their cephalothorax length (cm). (**B**) Scatter plot of Log_10_ dissolved oxygen consumption (mgO_2_.h^−1^) in relation to their Log_10_ body wet mass (g). (**C**) Scatter plot of the metabolic rate (mgO_2_.g^−1^.h^−1^) in relation to their body wet mass (g). Pink triangles: females individuals/blue circles: males individuals.

The body weight and size of individuals were highly and significantly correlated to oxygen consumption (Fig. 5A, Pearson coefficient *r* = 0.95, *P*-value <2.2e^−16^). The oxygen consumption of juveniles (R) can be deduced from their mass (M) according to the equation: R = aM^b^. In Log-Log space (Fig. 5B), “a” is the scale coefficient and “b” is the slope of the curve. Here, a = −0.8313 and b = 0.8239 (linear regression *P*-value <2.2e^−16^). This “b” factor of approximately 4/5 means that when the Log_10_ body weight increases by 5 g, the Log_10_ oxygen consumption increases by 4 mgO_2_.h^−1^. This means that even though the metabolic rate is divided by the body weight of the individuals, smaller animals nevertheless consume significantly more oxygen per gram of fresh matter than larger individuals (Fig. 5C, linear regression *P*-value = 1.46^−14^). Under the same conditions, 7-month-old juveniles weighing less than 20 g consumed 95% more oxygen per unit of fresh matter than juveniles weighing more than 80 g.

**Fig. 6.**
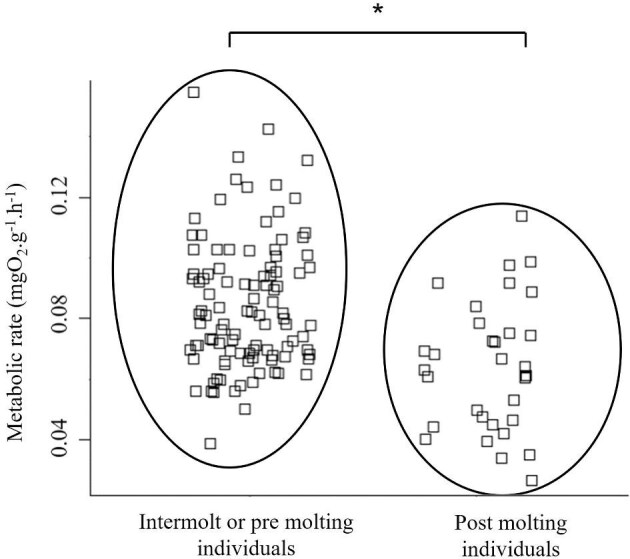
Strip chart of the metabolic rate (mgO_2_.g^−1^.h^−1^) of Mediterranean spider crab *Maja squinado* juveniles, according to their molting stage.

Also, as expected, 7-month-old juveniles have different metabolic rates depending on molting stage. Postmolt individuals (A-B molting stages) have a significantly lower oxygen consumption per g of fresh matter than other individuals that are most probably in molting stage C (intermolt) and, possibly, premolt stage (D0-D2) (0.064 ± 0.022 and 0.084 ± 0.024 mgO_2_.g^−1^.h^−1^ for postmolt and intermolt stages, respectively, *P*-value <10^−5^) (Fig. 6).

## Discussion

### Effect of the developmental stage on metabolic rate of *Maja squinado*

The RMR for each developmental stage of *Maja squinado* tested in this study (i.e., zoea 1, zoea 2, megalopa, first juvenile stage C1, 7-month-old juveniles, and adults) was set to a specific level, although it is normalized to the individuals' body mass. Data obtained revealed that zoea 1 larvae have a higher metabolic rate than zoea 2 individuals. This decrease during larval development has been observed in many decapods and could be explained, according to Anger (2001), by a disproportionate increase in metabolically inactive materials (fat, cuticle etc.). This decrease in oxygen consumption in *M. squinado* could also be due to their feeding mode: zoea 1 still draws energy from egg reserves, while exogenous food is the main energy source for zoea 2 larvae. This slight decrease in metabolic rate for zoea 2 could potentially save energy without risking reserve depletion. A difference in nutritional composition has already been shown between the zoea 1 and zoea 2 stages of *M. squinado* during the molt interphase (stage C). Indeed, Rotllant et al. (2014) have shown that the lipid to dry weight ratio of the zoea 2-C stage is almost three times that of the zoea 1-C stage, while the protein and sugar ratios are no different. Except for this decrease in oxygen consumption between the zoea 1 and zoea 2 stages, the metabolic rate in our study increases continuously from zoea 2 to the megalopa and first juvenile stages. This is in contradiction with what has been shown by Anger (2001) on other decapod species. This author suggests that in rare cases, the increase in metabolic rate at certain larval stages may be due to an increase in temperature sensitivity or a drastic change in feeding behavior. Indeed, access to food is a key factor in the metabolic rate of larvae. When energy intake is reduced, herring larvae negatively regulate their SMRs (Moyano et al. 2017). For most of the species included in these studies, the feeding rate decreased drastically as ontogeny progressed (Anger 2001; Gligorescu et al. 2019), which is not the case in our species: the megalopa stage of *M. squinado* is not a nonfeeding stage.

The exponential increase in metabolic rate in 7-month-old juveniles could be explained by growth intensification: with each molt, the size increment is around 30% (Guerao and Rotllant 2010; Durán et al. 2012). Larger individuals may therefore require more energy, as molting requires higher levels of calcification. It has already been hypothesized that the growth rate coefficient most probably includes the energetic effort required to search for and ingest the necessary amount of food, undeniably correlated with the individual's size: this is the “ecological metabolic cost of growth” (Peterson et al. 1999; Dmitriew 2011; Glazier 2015).

Young adults (7-month-old juveniles) have a much higher metabolic rate than larvae and C1 juveniles. Although the study temperatures differed (19°C for the early stages and 14°C for the 7-month-old juveniles), the difference between the two groups is so substantial (a 55-fold difference) that the comparison remains valid. Moreover, oxygen consumption increases markedly with temperature (Brown et al. 2004; Glazier 2015; Chabot et al. 2016). Therefore, it is highly likely—if not inevitable—that the oxygen consumption of juveniles and adults at 19°C would have been even higher, further accentuating the already significant difference observed. We can hypothesize that this change is mostly due to the development and complexity of organs, making essential biological processes (self-maintenance) more energy-consuming. This higher metabolic rate could also be due to the diversification and intensification of activities and behaviors, the latter being strongly correlated with metabolic rate.

However, a significant decrease in metabolic rate is observed between 7-month-old juveniles and adults (both studied at 14°C). As previously suggested, this could result from the fact that, in younger individuals, most of the metabolic rate is devoted to growth, whereas in adults it is primarily associated with self-maintenance processes (Parry 1983). It could also be attributed to very different survival strategies and behaviors (Biro and Stamps 2010). This type of ontogenetic changes in behavior and locomotor activity (from exploratory to sedentary behavior, or the contrary) has already been observed in some species, such as *Solea solea* (Durieux et al. 2010), *Dentex dentex*, and *Sciaena umbra* (Ducos et al. 2022). In *M. squinado*, juveniles are almost immobile (González-Gurriarán and Freire 1994; Hines et al. 1995), feeding on algae and nearby invertebrates and using an active camouflage strategy to avoid numerous predators. Adults, on the other hand, have few predators and only use passive camouflage, and their locomotor activity can be strongly increased. It is common for adults to move around (bathymetric migrations) during the reproduction period. Typically, adult *M. squinado* ascend from depths of up to 200 m to surface waters at 10 m to reproduce and lay their eggs (González-Gurriarán and Freire 1994), much like their sister Atlantic species *M. brachydactyla*, which can travel more than 500 m in 5 days at the time of migration, while juveniles travelled less than 10 m over the same time (Hines et al. 1995). It is therefore likely that juveniles have a RMR closer to their maximal metabolic rate (low aerobic scope), whereas adults need a larger “energy reserve” (Pettersen et al. 2016; Prinzing et al. 2023). This aligns with the “compensation” hypothesis, which suggests that individuals with a low metabolic rate, and thus low self-maintenance costs, would have more energy to devote, for example, to reproduction (Blackmer et al. 2005; Burton et al. 2011). Furthermore, metabolic rate has a significant impact on fitness depending on the environment. For example, in the European sea bass, *Dicentrarchus labrax*, individuals with a high metabolic rate tend to lose more body mass in a food-limited environment than individuals with a lower metabolic rate (Killen et al. 2011). In our species, adults spend most of the year (September–May) at depths of over 80 m in a much more food-limited environment than juveniles (which are at depths of 10–20 m). Therefore, a low metabolic rate could be an advantage for the former, with possibly a better survival rate. This has also been shown in fish, for example, the brown trout *Salmo trutta* (Álvarez and Nicieza 2005) and in mammals, for example, the North American red squirrel, *Tamiasciurus hudsonicus* (Larivée et al. 2010), where individuals with a lower metabolic rate relative to their body mass maintain their reserves and survive the winter season better than those with a higher metabolic rate.

### Impact of endogenous factors on the metabolic rate of 7-month-old juvenile and adult *Maja squinado*

Adult male *M. squinado* have a metabolic rate that is twice as high as that of adult females, while this difference is not observed in 7-month-old juveniles that are not yet sexually mature. Sexual differentiation therefore has a direct impact on individual metabolic rate level. This difference has already been observed in the Atlantic species *M. brachydactyla* (Cerezo Valverde et al. 2009). The difference between adult males and females could be due to behavioral differences, as males are much more competitive than females for territory, and especially for reproduction. It has been established in many decapod species that while males show dominant aggressive behavior, females tend to be submissive (Pinheiro and Fransozo 1999; Turra 2005) and may be less selective in their choice of mate (Christy and Salmon 1991). Females may even stop feeding for several days until their eggs hatch (Christy and Salmon 1991). Typically, it has been observed in various species that individuals with a high metabolic rate are often dominant and defend a larger feeding territory than those with a low metabolic rate (Biro and Stamps 2010). Additionally, in *M. squinado*, since females do not compete with each other for reproduction, they may prioritize energy conservation before gestation. For example, in the terrestrial arthropod *Nauphoeta cinerea*, metabolic rates before mating in females are inversely correlated with gestation duration (Schimpf et al. 2012). This difference in metabolic rate may not be permanent and could change depending on the reproductive cycle. In the case of *Maja brachydactyla*, for example, while adult locomotor activity is very low in summer and autumn, with similar levels to those observed in juveniles, this activity increases drastically in September–November as they return to deeper water (Hines et al. 1995). This difference in activity according to the reproductive cycle could have a significant effect on their metabolic rates. It would therefore be interesting to monitor the metabolic rates of males and females throughout the reproduction period.

The data obtained in this study demonstrate that despite adjusting oxygen consumption to the individuals' weight to calculate the RMR, it remains, for the 7-month-old juveniles, twice as high in smaller individuals (<20 g) than in larger ones (>80 g). This has also been demonstrated in the Atlantic sister species *M. brachydactyla* (Cerezo Valverde et al. 2009) as well as in many other species ranging from plants to animals (Kozłowski et al. 2003; Da Silva et al. 2006; Killen et al. 2010; Glazier 2014; Glazier and Gjoni 2024). This difference is partly due to the fact that smaller individuals have an increase in ATP turnover, mitochondrial density and proton leakage, shortening their physiological times (Da Silva et al. 2006). In our study, the logarithm of routine oxygen consumption (mgO_2_.h^−1^) of 7-month-old juveniles maintained at 14°C varies with the individual mass (g) by a factor of 0.82, which corresponds to what was found by Cerezo Valverde et al. (2009) for the Atlantic sister species, *M. brachydactyla* (“b” = 0.847 at 18°C, from juveniles to adults). This “b” factor generally varies between 2/3 and 1 between and within species, depending on various endogenous and exogenous factors. It has been shown that the value of “b” factor is closely linked to the ecological lifestyle of the species. For example, in pelagic teleosts, it is close to 2/3 and is equal to 0.78 and 0.80 in bentho-pelagic and benthic teleosts, respectively (Killen et al. 2010). This is consistent with the value obtained for the benthic *M. squinado* juveniles. However, studies have also shown that higher activity leads to increases in b, approaching the value of 1 at high activity levels (Glazier 2014). In the present study, spider crabs are rather calm and display camouflage behavior despite a “b” factor approaching 1. However, we only tested the RMR of individuals; perhaps the measurement of their maximum metabolic rate would reveal an even greater increase in b. However, this *b* value should be interpreted with great caution, as numerous studies have shown that it can vary widely—from 0.1 to 1.6—across different clades and under certain conditions, being influenced by a range of internal and external factors (Glazier 2022).

Seven-month-old *M. squinado* juveniles have a CL that ranges from less than 3 cm to more than 9 cm, despite identical rearing conditions. These individuals were raised in the same tank. Growth difference may be due to food and/or social competition. Organisms exhibit a certain metabolic plasticity, with different growth rates in response to changes in resources such as food (Brown et al. 2004). It has also been observed that aquatic organisms can adapt their behavior by choosing their environment (such as modifying temperature) or by altering their activity levels to manage their growth rates (Evans 1998). In our study, smaller individuals could hide in narrow artificial shelters, making them unreachable. This could give them an advantage in terms of survival by avoiding intraspecific predation (cannibalism), which is a common behavior for this species. Reduced growth for some individuals may therefore be genetic, chosen (behavioral), or imposed (competition).

Molting has a major impact on the individual metabolic rate. Postmolt individuals (stages A, B) have a much lower metabolic rate than intermolt or premolt animals (stages C and D, respectively). This is consistent with data obtained on the American lobster, *Homarus americanus* (Penkoff and Thurberg 1982). Locomotor and feeding activities significantly decrease or even cease during molting in crustaceans, remain low in postmolt animals, and only begin to increase again at stage B2 or C1, reaching their maximum during intermolt (Lipcius and Herrnkind 1982; O'Halloran and O'Dor 1988). In the Caribbean spiny lobster, *Panulirus argus*, social behavior also changes during molting, with individuals showing submissive and avoidance behavior toward their counterparts, probably in order to avoid cannibalism (Lipcius and Herrnkind 1982). Metabolic rate therefore seems to follow this pattern, with a minimal reduction during molting and postmolting since the animal draws energy solely from the reserves of its digestive glands, and a maximum increase occurring during intermolt, that is, the phase of active foraging, exocuticle mineralization, tissue growth, and preparation for the next molt. Huang et al. (2015) also showed in the Chinese mitten crab, *Eriocheir sinensis*, that genes expressed during intermolt were specific to energy storage (glycogenesis and fatty acid pathways), while genes expressed in post-molt were related to carbohydrate, neutral lipid, and amino acid metabolic processes (notably glycogenolysis pathways).

## Conclusion—perspectives

The Mediterranean spider crab, *Maja squinado*, an endangered species protected by Annex 3 of the Barcelona Convention, is in sharp decline (Durán et al. 2012, Marengo et al. 2023) despite efforts to regulate its fishing (United Nations Environment Program 2019). However, it can be reared in inland facilities and appears to be an excellent model for exploring physiological parameters such as the impact of growth, size, sex, and molt on RMR as a proxy for energy use at the individual level. To our knowledge, this is the first study to focus on *M. squinado* metabolic rates using different ontogenetic stages (zoea 1, zoea 2, megalopa, C1 crabs, 7-month-old juveniles, and adults). The significant changes in energy use observed through development are likely due to differences in feeding strategies and internal body composition (zoea 1, zoea 2, megalopa, and C1), as well as differences in the energy cost of growth (larvae vs. juveniles) and/or differences in behavior and locomotor activity (juveniles vs. adults). Individual metabolic rates were also dependent on intrinsic factors within each developmental stage. A size effect occurs between individuals in juveniles, with a metabolic rate that is twice as high in small individuals (<20 g) than in large ones (>80 g). In addition, a reduced oxygen consumption is observed in postmolt individuals that stop feeding, showing much lower levels of locomotor activity (Lipcius and Herrnkind 1982; O'Halloran and O'Dor 1988). In breeding adults, males have a metabolic rate that is twice as high as that of females, often reflecting dominance behavior (Biro and Stamps 2010).

Altogether, these results indicate that it is necessary to take into account key biological factors such as life stage, reproductive cycle, individual size, and molting stage in order to assess individual performance. This should be considered in order to optimize the conditions for individual release into the natural environment for viable ecological restoration programs. For this study, knowledge of metabolic rates, particularly in early life stages, has highlighted several key points. For instance, that the molting period is particularly unsuitable for individual release into the natural environment, and that the energetic cost of growth of 7-month-old juveniles may be incompatible with prolonged maintenance under rearing conditions (e.g., individual cages, frozen food, etc.). These findings suggest that releasing intermolt crabs at younger stages (C3–C5, just a few weeks old), may be a more suitable strategy.

Further studies will be needed, particularly to assess the influence of external factors such as environmental temperature and day–night cycles, for example.

## Data Availability

The authors commit to publishing the data associated with this manuscript on the official French government website: recherche.data.gouv.fr.
